# Beyond Academia – Interrogating Research Impact in the Research Excellence Framework

**DOI:** 10.1371/journal.pone.0168533

**Published:** 2016-12-20

**Authors:** Emma Terämä, Melanie Smallman, Simon J. Lock, Charlotte Johnson, Martin Zaltz Austwick

**Affiliations:** 1 UCL Institute for Sustainable Resources, University College London, London, United Kingdom; 2 Department of Science and Technology Studies, University College London, London, United Kingdom; 3 Centre for Advanced Spatial Analysis, University College London, London, United Kingdom; Lancaster University, UNITED KINGDOM

## Abstract

Big changes to the way in which research funding is allocated to UK universities were brought about in the Research Excellence Framework (REF), overseen by the Higher Education Funding Council, England. Replacing the earlier Research Assessment Exercise, the purpose of the REF was to assess the quality and reach of research in UK universities–and allocate funding accordingly. For the first time, this included an assessment of research ‘impact’, accounting for 20% of the funding allocation. In this article we use a text mining technique to investigate the interpretations of impact put forward via impact case studies in the REF process. We find that institutions have developed a diverse interpretation of impact, ranging from commercial applications to public and cultural engagement activities. These interpretations of impact vary from discipline to discipline and between institutions, with more broad-based institutions depicting a greater variety of impacts. Comparing the interpretations with the score given by REF, we found no evidence of one particular interpretation being more highly rewarded than another. Importantly, we also found a positive correlation between impact score and [overall research] quality score, suggesting that impact is not being achieved at the expense of research excellence.

## Introduction

### Background

The wider impact of research is becoming an important concern across higher education in the UK, where it is being seen as a key component of universities’ societal and economic role [1]. In particular, the increasing need for publicly funded research to be more accountable to the taxpayer and treasury has led to a large-scale attempt to capture the value and benefits of research and to build this into the national research evaluation and funding process via the Research Excellence Framework (REF) [2].

To assess the quality of research at universities, the Higher Education Funding Councils (e.g.for England: HEFCE) introduced the REF in 2014 [3] to replace the previous Research Assessment Exercise (RAE) of 2008 that had been on-going since 1986. REF evaluates three elements with different weightings: 1. Originality, significance and rigour of research outputs (65%); 2. Reach and significance of impact (20%); and 3. Vitality and sustainability of research environment (15%). The impact (2.) measure was new for 2014 and is a major distinction between the REF and its predecessor, the RAE [4].

For the first REF assessment in 2014, institutions made submissions to 36 distinct units of assessment (UoAs) based on areas of research. The submissions were assessed by an expert sub-panel for each UoA, working under the guidance of four main panels, A-D, based on broad areas: Panel A focused on the life sciences, Panel B on the engineering and physical sciences, Panel C on the social sciences and Panel D on the arts and humanities (see S1 Table for list of panels and units of assessment within the panels).

Evidence of impact was provided to the REF exercise in the form of impact statements or case studies, which followed guidelines and a template provided by the REF, including descriptions of the research and the process by which impact was achieved beyond academia. The comprising sections were: 1. Summary of the impact, 2. Underpinning research, 3. References to the research, 4. Details of the impact, and 5. Sources to corroborate the impact. These case studies were then evaluated by expert sub-panels. The REF process advised (through guidelines and people associated with the process) that all types of impact beyond academia were admissible, but that the impact had to benefit a particular sector and include beneficiaries outside of the academe as well as link directly back to published academic work of reasonably high international standard (‘two-star’ minimum) [5] (p.29, para 160b). Furthermore, each of the REF panels provided criteria by which submissions would be assessed [6]. These criteria included examples of impact.

This new inclusion of impact has triggered academic debates around the impact of research in much too broad terms to cover here substantially. However, we would like to mention, in particular, the discussions around the moral imperative to ensure impact [6]; the potential for enhanced partnerships, co-production or boundary work between practice and academia [7,8,9]; ensuring equitable, socially accountable impacts of knowledge [6,7,8,9]; balancing the cost and gains from impact assessment [10,8,11]; whether it is creating pressures to change the character of knowledge [6,7,12,2]; and, possible threats/gains to academic freedom and intellectual autonomy [7,13,11,9,14]. Further topics of discussion include (but are not limited to) the challenge of attribution of impact [10], also in part stemming from the time lag between publication of research results and implementation through policy or practice [7,12,2] and the different definitions and dynamic nature of impact itself [10,12].

### Definition of impact: it varies

The concept of research impact is not new. In general terms, research impact is clearly important: it is commonly recognised as an inherent and essential part of research, and an important way in which publicly funded research is accounted for [15,16,17,18,19,20]. Impact implies a relationship through which researchers gain funding in return for contributing to society, through activities such as knowledge sharing via public or stakeholder participation or commercial exploitation of a new finding or technique, for example.

The word ‘impact’, however, has slightly different connotations in different contexts, including within the recent REF. Currently used definitions of impact largely originate from individual UK research council guidelines for funding applications and HEFCE guidelines for REF2014 [3]. Oancea [21] previously described how understanding of impact was subject specific and, in keeping with that, within the REF2014, each assessment panel (A-D) had slightly different definitions embedded in their guidelines. For example, while examples such as impacts on society, culture and economic prosperity were common to the guidelines for all panels, Panel A had a specific example around ‘impact on production’, while Panel D mentioned ‘Education’. [6]

In addition, individuals, research communities and research institutions have their own conception of impact as it pertains to their specific context: impact to a mathematician is different from that of a medical researcher or sociologist. We therefore continue the discourse started by Oancea [21] on subject-specific understanding of impact.

It is however important to note that the types of impacts presented in the REF can only be a rough proxy for how researchers view impact. Impact, and indeed the impact agenda, is much broader than REF impact alone. For the purposes of this research however, we deal with 'impact' as an actor's category, based on the definition and guidance given by the REF process. Indeed it is part of our objective here, to understand more fully how actors (researchers and the REF) interpret and validate the term 'impact'.

### Assessing impact

Studies looking at the procedures and methodologies to investigate individual, programme and institute-wide impact of research are becoming more common [22,23,24,25,26]. Already the REF pilot exercise [27,28] highlighted that gathering evidence on research impact is a new endeavour with little institutional infrastructure available to support the process, and a heavy reliance on the personal knowledge of senior academics. Given the broad ways in which impact is interpreted, it is not surprising that the possibility of reviewer bias towards personal experience over ‘untested guidelines’ has also been raised [14,29,30,31,32].

The increased importance of impact and its new relationship with research funding allocation has meant that impact can be a controversial topic in the academic research community. This is particularly so in research areas that are not traditionally viewed as having obvious or immediate impact–for instance in the less applied fields of research such as fundamental natural science and certain humanities branches. Other fields do, however, have strong traditions of impact, such as the bio-medical ‘bench to bedside’ translational research, and the social sciences via economic and social policy. We address this by reviewing impact outcomes of the REF2014 by discipline (UoA) as well as by institution.

To date, three previous studies have examined the REF impact data: Firstly, RAND Europe was commissioned by HEFCE [33] to examine the process of assessment of submissions by panels of academics and research users. Looking at the strengths and weaknesses of the assessment process and whether or not it delivered reliable and fair outcomes, the study concluded that a large majority of the academics and research users on the panels felt that the process enabled them to assess impact in a fair, reliable and robust way.

Secondly, RAND Europe also evaluated universities’ preparation of submissions describing impact [34]. Looking at a sample of 21 HEIs and three stakeholder groups that included HEI leadership teams, HEI case study authors and research users, the study set out to understand their experience with the impact submission process. They found that while the process helped HEIs to develop skills in understanding and identifying impact, which they argued was starting to move towards a greater focus on impact in HEI research, complying with the impact component of the REF was found to be burdensome and resource intensive.

Thirdly, Digital Science, a division of Macmillan Science & Education in conjunction with the policy institute at King’s College, London [34] was also commissioned by HEFCE to look at the nature, scale and beneficiaries of research impact (hereafter the ‘King’s study’). They used a text mining approach (MALLET) to look at the 6,679 non-redacted impact case studies in order to identify general patterns and thematic structures in research impact across the sector. They found that research impact was wide and diverse, identifying 60 unique ‘impact topics’. They also found 3709 unique ‘pathways to research’, which the authors argued demonstrated how the sources of impact were also diverse. And while this might not have been the main objective of the research, some of the topics identified in this study however were difficult to understand as ‘types’ of impact and seemingly represented orthogonal categorizations: alongside topics such as ‘technology commercialization’ and ‘informing government policy’ were other topics such as ‘climate change’, ‘crime and justice’, ‘Asia’ and ‘cancer’, with limited exploration of the classification ontologies these terms represent. Similarly, the analysis also did not relate the topics to one another, making it difficult to draw many conclusions beyond acknowledging the diversity of impact, leaving questions such as whether particular interpretations of impact were more highly rewarded than others unanswered by this analysis.

This paper aims to build on the insights gained from these previous studies and to contribute further to critical reflection. Firstly, we look beyond the REF assessors’ views of impact (RAND Study [33]), to examine researchers’ and institutions’ interpretations of impact–both across the whole of the REF and within sample universities. Secondly, by taking an alternative text mining approach, we extend the understanding gained in the King’s study [34], to build a sharper overview of the impact landscape. In particular, we set out to understand the relationship between types of impact and reward, as well as between impact and research excellence.

### Measuring impact

In this paper, we compare institutional REF2014 outcomes to the entire database, and examine one institution in detail, namely UCL. We believe UCL is a good case study for looking at impact for a number of reasons: Firstly, as internal researchers, we were able to access some of UCL’s redacted material and as such have been able to look at and comment upon the whole range of material submitted to the REF not just that which is publicly available. Secondly, UCL covers a diverse range of research disciplines and submitted 91% of its eligible staff to the REF process (some Universities submitted as few as 7% of eligible staff). This means the UCL results are representative of the institution as a whole.

## Data & Methods

### Data

We used five sources of data for the research presented in this paper. The data for all institutions’ impact case studies was obtained from the REF2014 results portal [35] where the 6637 impact case study submissions into the REF evaluation process are accessible. Secondly, we looked at seven of the institutions that the Times Higher Education Supplement listed as top-ranking institutions in terms of their impact outcome (namely Cardiff University, Institute of Cancer Research, Imperial College London, King’s College London, London School of Hygiene and Tropical Medicine (LSHTM), St George’s and UCL). These were chosen to reflect a range of specialist/versus broad focused universities, those with and without medical schools and those in London and outside London, in order to check that these factors were not having a disproportionate effect on the results. Thirdly we looked at the 283 UCL impact case studies available from the REF online database. Fourthly, the outcomes of the peer evaluation (results) for UCL and others (also available on the portal). Finally, the guidelines for submissions to REF, available at http://www.ref.ac.uk/pubs/2012-01/ [35,3].

To prepare the data for the Iramuteq analysis described below, it was necessary to format it for input. The code used to do this has been made freely available online at https://github.com/martinaustwick/REF_analysis_text_preparation/blob/master/REF_Impact_cleaner_inc_UCL.ipynb, created using the Python programming language (and using the Pandas library for data manipulation). This is presented in a Jupyter Notebook (previously iPython) and includes annotations to provide context to the code. In brief, the process begins by iterating through each UoA. For each UoA, the code iterates through every entry, gathering the data for each in the JSON format (JavaScript Object Notation) from a REF API (Application Programming Interface) call, and structuring it into a Pandas dataframe. This is a programming framework that allows our Python code to request a specific dataset from the REF database, and we are using UoA as our filter, to reduce the quantity of data interrogated at each step. Every entry is then processed, creating asterisked references for title, UKPRN, UoA, ID, and panel, which is required for the main analysis stage. The text associated with the fields “Impact Summary” and “Impact Details” are then pre-processed. This pre-processing consists of removing a series of characters that interfere with Iramuteq parsing ("*", '(', ')', '\'', '\‴, '[', ']', '{', and '}'), changing currency values to words (e.g. “$” is converted to “dollars”), removing URLs, and converting all words to lower case. Processed text for Summary and Details are concatenated with the asterisked identifiers (see above) with appropriate whitespace. The processed text of each of these entries (with identifiers) is combined and saved into one large (46MB) text file that forms the basis for further analysis. Conditional filtering on UKPRN allows us to easily select or exclude individual institutions for special attention prior to saving to text file; thus institution-specific text files can easily be generated.

### Method

To look at how researchers interpreted impact in their case studies, a computational text mining technique was used to examine the discourses within the impact case studies submitted. As described above, sections 1. ‘Summary of impact’ and 4. ‘Details of impact’ were selected for analysis. The analysis was carried out on three levels: 1) the entire impact case study database; 2) the UCL case studies and a selection of six other top-ranking institutions separately, chosen to reflect varying geographies and institutional focuses; and 3) the UCL submissions to each REF panel (A-D) separately. Altogether we aimed for these different depths of analysis to inform how the interpretation of impact differed by e.g. discipline, size of database or location (central London vs. rest of the country).

The IRAMUTEQ (*Interface de R pour les Analyses Multidimensionnelles de Textes et de Questionnaires*) software package was used to analyse the corpus of text derived from the impact case studies [36]. The tool produces a statistical map of a corpus, based on the pattern of words within the corpus: the resulting simplified pattern, or ‘map’ is based on the Word Space Model [37], which is a computational model that derives the meaningful relationships and associations between words from the way in which they are distributed and situated [38]. The map reflects significant clustering of language, as well as meaningful links from individual words to clusters. IRAMUTEQ selects the ‘meaningful’ words in the corpus by reducing them to their root forms (lemmatizing), then dividing them into function words (articles, prepositions and pronouns) and ‘content’ words (nouns, adjectives, verbs and adverbs) by grammatical filtering. Only the content words are used in the analysis, although the function words are retained for context (e.g. sample sentences).

The software then breaks the corpus into text segments of fixed length, mimicking sentences within the text. The presence or absence of a particular word in each text segment is then mapped into a contingency table and this contingency table is used to group the words in the corpus into classes according to their distribution in the text, grouping together words which are used in similar ways. Descending Hierarchical Classification (DHC) is used to create these classes. Beginning with all of the words in one class, DHC iteratively splits the full word list into two classes, then splits the biggest of these two into another two, and so forth. Each split is made by considering all of the possible ways that the classes could be cut into two and accepting the division that produces two classes which are the most dissimilar to each other, according to a chi-squared criterion. This continues until ten (default) rounds of splitting has been undergone, or until no further splits can be made (a setting of eight, ten and twelve rounds was tested with sample data, and ten was not found to be a limiting factor).

Finally, the classes produced are cross-tabulated with the words in the corpus as part of a correspondence analysis, which presents the contingency table as a graph showing the frequency and relationship of each word to the relevant classes. At this point, any metadata used to tag the corpus (in this case unit of assessment; panel; institution) is mapped onto the classes, so it is possible to see which UoA etc. are most closely associated with each class. Further details of the software and statistical analyses involved are provided in the references [39,40,41,42,43, 44].

The differences between the algorithms that IRAMUTEQ (analogous to ALCESTE) and MALLET (used in the King’s study) are based upon are explained in detail in Bholat et al [45]. In summary, MALLET uses Latent Dirichlet Allocation (LDA) which is a mixed-membership model in which words and documents are assigned probabilities related to multiple topics. For each word in each document a topic assignment is made and the probability of that word appearing in that topic calculated. The algorithm therefore looks for similarity and gives a weighting for each topic, ultimately identifying prevalence of topics. IRAMUTEQ in contrast uses Descending Hierarchical Classification (DHC) which tries to discover stable classes of terms that are maximally associated within classes while being minimally associated with other classes–effectively searching for difference and creating classes that are as different to each other as possible. While the user specifies the maximum number of divisions to take place, the process will stop before that if there are no further statistically significant divisions possible. Overall, this approach provides an alternative lens to look at the data. In particular, this descending approach allows the relationship between classes to be understood. Table 1 provides a brief comparison of the King’s method to ours.

**Table 1:** Comparison of the text mining methods used in the King’s [34] vs. UCL analysis.

	King’s	UCL
**Software**	Natural Language Toolkit 3.0 (MALLET)	Iramuteq 0.7 alpha 2
**Programming language**	Python	R
**Topic modelling method**	Latent Dirichlet Allocation (LDA)	Descending Hierarchical Clustering (DHC)
**Generative technique**	Finding similarities	Finding differences
**Website**	http://www.nltk.org/	http://www.iramuteq.org/
**Reference**	Bird S, Loper E, Klein E. *Natural Language Processing with Python*. 2009. O’Reilly Media Inc.	Ratinaud P. IRAMUTEQ: Interface de R pour les Analyses Multi-dimensionnelles de Textes et de Questionnaires [Computer software]. 2009.

### Interpretation of impact and quality

Once the vocabulary was grouped into classes via IRAMUTEQ, the salient themes of each class were identified. To do this, each co-author (representative of a wide range of disciplines) individually reviewed the sources of information produced by the software (classes, correspondence analysis etc.) to determine a (possible) interpretation for each of the classes, which was negotiated to agree on the most plausible interpretation for each class. A consensus was reached on the choice of ‘titles’ for each class. Finally, the resulting topics were cross-referenced twice independently with the language present in the REF2014 guidance documents on criteria and working methods by panel [3,35].

To understand whether impact affected research quality, the institution-specific UoA impact scores (Impact GPA) were investigated in relation to the research quality scores (Output GPA) and any correlations identified. For this analysis the entire results database of 154 institutions and their UoA submissions was used comprising altogether 1911 data points: detailed breakdowns of sector performance has been made available by unit, panel and Higher Education Institution (HEI) by REF; each unit at each university is listed, with the proportion of submissions achieving 4,3,2 and 1 ‘star’ ratings. From this a grade point average was produced via a simple weighted sum of these percentages:

$$GPA={\Sigma_{i=1}}^{4}p_{i}i$$ (1)

where *p*_*i*_ is the proportion of submissions being rated at *i* star quality, and runs from 1 to 4.

## Results and Interpretation

The analysis of the entire results database (6637 impact case studies) resulted in six classes or interpretations of impact type: Education, Public engagement, Environmental and energy solutions, Enterprise, Policy and Clinical uses. As explained above, this was the maximum number of statistically significant classes that IRAMUTEQ was able to identify. The subjects of the classes reflect the majority of the categories outlined in the REF guidelines, although impact on law and international development were significant omissions. If these impacts were present in the REF submissions, they were not present in sufficient quantity to be picked up during this analysis. We will discuss this further when we take a closer look at analysis of the individual panel submissions.

As we have explained above, to look at this in more detail and to understand the relationship between types of impact and other factors measured in the REF, we focused our future attention on the UCL data. So that we could be confident that we were not focusing on a case study that was a-typical however, we also ran the analysis separately for six other of the ten top-ranking institutions (according to Times Higher Education in terms of their impact outcome), namely Cardiff University, Institute of Cancer Research, Imperial College London, King’s College London, London School of Hygiene and Tropical Medicine and St George’s. These represented a range of geographies and degrees of specialism, to act as comparisons to the UCL data—Cardiff University is a broad-based institution much like King’s and UCL. London’s Institute of Cancer Research is a leading institution in clinical medicine and biological sciences. Imperial College London is best known for its focus on engineering, technology and medicine. The London School of Hygiene and Tropical Medicine (LSHTM) focuses on public health and the environment, and St George’s (also in London) focuses on medical sciences and population health.

The 283 UCL impact case studies produced five distinct classes, one less than the entire database analysis. A summary and comparison of the entire database and UCL classes is given in Table 2. Details of the UCL data are given in Table 3, and the entire database and additional institutions analysis is in S2 Table. Comparing the seven institution-specific analyses with the entire database, the institutions whose classes most closely corresponded to those produced by the analysis of the while database was UCL. We therefore argue that UCL makes an interesting reflection of the sector as a whole due to its broad base of faculties and REF submission. The key difference between the classes produced in the UCL analysis and those of the whole database was the absence of a class around environmental and energy solutions (Class 3). The subject matter of this class was, however reflected in the subsequent panel–specific analysis (described below). Going back to the original text, it would appear that the vocabulary relating to this class is distributed between UCL Class 1 (policy) and UCL Class 5 (enterprise). Many of the other high-ranking institutions analysed were more subject-specific, which is also reflected in their key areas of impact. We therefore argue that the UCL data provides sufficient breadth coupled to excellent impact to probe into further details.

**Table 2:** Comparison of Classes produced by IRAMUTEQ analysis of entire REF impact database of case studies and UCL impact case studies.

Classes produced in analysis of entire REF database	Classes produced in analysis of UCL impact case studies
***6637 texts*. *112531 words*, *87%w classified***	283 texts. 21952 words, 74%w classified
**Class 1**	**Class 4**
**(22.81%)**	(14.4%)
**Education**	Education
**Class 2**	**Class 2**
**(16.96%)**	(16.8%)
**Public engagement**	Public engagement
**Class 3**	
**(17.66%)**	
**Environmental and energy solutions**	
**Class 4**	**Class 5**
**(11.8%)**	(21.6%)
**Enterprise**	Enterprise
**Class 5**	**Class 1**
**(17.09%)**	(23.5%)
**Policy**	Policy
**Class 6**	**Class 3**
**(13.68%)**	(23.7%)
**Clinical uses**	Clinical uses

**Table 3:** Classes produced in the analysis of UCL impact case studies (all panels A-D)

Class and interpretive label	Ten most associated words	Typical sentence	Most associated REF Panel
**Class 1** (23.5%): Government policy, scientific evidence and management	policy, local, plan, government, authority, research, health, national, strategy, practitioner	*“the principal* ***contribution*** *of this* ***programme*** *of* ***work*** *has been to* ***provide*** *an* ***evidence*** *base for* ***informed*** *decision making at* ***local*** *and national* ***policy*** *levels regarding the configuration of acute* ***services*** *for severely mentally ill adults”*	Panel C–Social Sciences
**Class 2** (16.8%): Public engagement and arts	museum, public, audience, exhibition, history, event, gallery, art, film, visitor	*“we have also* ***worked*** *closely with the jeans for genes campaign in schools and at other fundraising* ***events*** *speaking and* ***appearing*** *in* ***films*** *to raise* ***awareness*** *of our* ***work***. *Our* ***work*** *has been* ***presented*** *as* ***part*** *of a living* ***exhibition*** *to* ***promote*** *the* ***public*** *understanding of* ***science****”*	Panel D–Humanities
**Class 3** (23.7%): Clinical applications	patient, treatment, clinical, disease, trial, therapy, treat, cancer, drug, diagnosis	*“for example in the* ***year*** *ending 2011 our* ***nhs*** *genetics laboratory* ***performed*** *diagnostic* ***tests*** *for* ***genetic*** *deafness* ***disorders*** *in over 1 100 uk* ***patients*** *and we provided both* ***molecular*** *and* ***clinical*** *input to reports”*	Panel A–Life Sciences
**Class 4** (14.4%): Education	school, education, teacher, IOE, child, curriculum, pupil, parent, study, young	*“while all* ***age*** *groups were evenly represented several* ***schools*** *benefitted particularly from the* ***provision*** *of guided* ***educational*** *tours for* ***year*** *6* ***pupils*** *age 10 11 to* ***support*** *the national* ***curriculum*** *of ww2* ***studies*** *as* ***well*** *as tours for* ***students*** *in continuing* ***education*** *and civics”*	Panel C–Social Sciences
**Class 5** (21.6%): Enterprise	company, corroborate, product, technology, customer, industry, detail, market, business, contact	*“****companies*** *have* ***invested*** *heavily in the* ***development*** *of* ***products*** *relating to these* ***technologies*** *and in a competitive* ***environment*** *have* ***included*** *these* ***products*** *in the* ***development*** *of their* ***business*** *strategies”*	Panel B–Physical Sciences

The five UCL classes were drawn from across the corpus, although certain panels and UoAs were associated with some classes more than others. Significant sentences from the original text were retrieved, examples of which are given in Table 3: classes with the most plausible interpretive labels; the ten most associated words, and; illustrative quotes from the original text to help explain the content more vividly. We also give the panel that is most associated with each class.

### Rewarding impact

**Fig 1:**
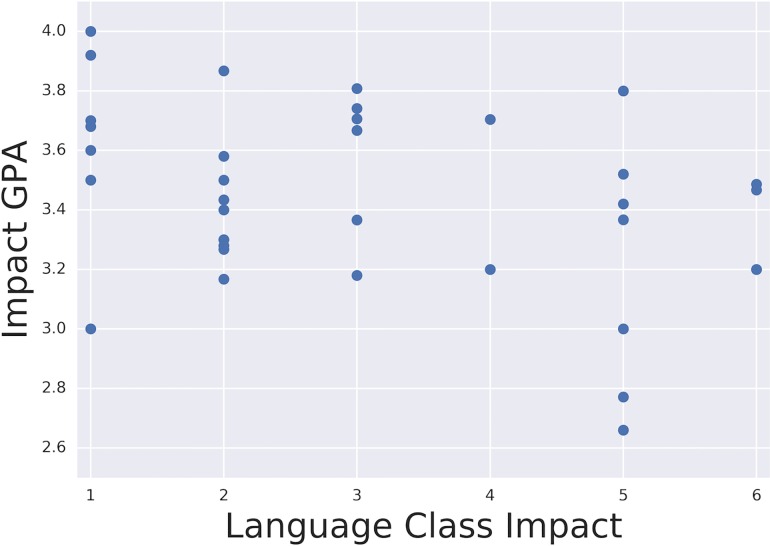
Impact GPA by Unit of Assessment vs. impact Class.

To understand which (if any) of the interpretations of impact or impact classes scored most highly, we again focused on the UCL case studies. The impact case studies were categorised according to the class that their unit of assessment was most closely associated with (see Fig 1) and the impact score mapped onto them (Fig 1). Most submissions were strongly associated with one class only. Some submissions however contributed to more than one class–in other words covered more than one interpretation of impact. In order to check that these less focused interpretations of impact didn’t affect the impact score, we placed these cases into a separate category. From Fig 1 it is possible to see that impact scores were spread across the range of UoAs (and therefore panels), with no one UoA-specific interpretation of impact standing out as gaining on average a higher score than another–even when separating out those which cross more than one interpretation of impact. We therefore argue that there does not appear to be any particular interpretation(s) of impact that has been more highly rewarded than another, and submissions which evince more than one form of impact are not advantaged (or disadvantaged).

### Association of classes by panel

The King’s study suggested that different types of impact are common in different disciplines. To test this hypothesis and to explore this link further, we carried out the same text analysis but looking at the UCL data submitted to each of the REF panels separately.

We found that while most panel submissions are dominated by one or two impact classes, they all produced classes relating to policy, evidence and management. Importantly, the classes produced appear to correlate to the subject focus of the panels themselves, see Tables 4–7. For instance, classes on education, arts and museums were absent from the analyses of Panels A and B, despite ‘culture and creativity’ being an example given in the impact guidelines for both these panels. Similarly, Panels C and D produced no classes relating to health or clinical practice, even though Panel C giving ‘impact on health and welfare’ as an example of impact in their guidelines. And although impact on the economy was given as an example in all of the panels, only Panel B exhibited strong impact in the area of enterprise / business. This suggests that impact was being interpreted by the researchers and their institutions and not just followed from the guidelines.

**Table 4:** Panel A–Life Sciences

Class and interpretive label	Ten most associated words	Typical sentence
**Class 1** (48.16%): Public health policy	health, service, programme, policy, train, department, public, report, national, evidence	*“the screening* ***service*** *covers every* ***london*** *borough and* ***supports*** *public* ***health*** *england phe to* ***manage*** *outbreaks of* ***tb*** *nationally our subsequent evaluation of the expanded* ***service*** *contributed to* ***decisions*** *for* ***nhs*** *to take over the* ***funding*** *of the* ***service*** *which is now hosted by uclh on* ***behalf*** *of* ***london****”*
**Class 2** (23.13%): Clinical applications and interventions	diagnosis, image, disease, test, brain, genetic, technique, glaucoma, diagnostic, gene	*“****new*** *diagnostic* ***tests*** *for* ***genetic*** *deafness have been* ***introduced*** *and healthcare guidelines and professional standards adopted through our* ***investigation*** *of the aetiology of childhood* ***onset*** *hearing loss* ***disease*** *prevention has been achieved by our research on antibiotic associated* ***deafness****”*
**Class 3** (28.71%): Clinical trials and treatment	treatment, patient, treatdose, save, phase, trail, regimen, relapse, transplant, cost	*“assuming 550 liver* ***transplants*** *per* ***year*** *in the uk since 2008 we can* ***estimate*** *that with 90 of* ***patients*** *treated with* ***tacrolimus*** *and 10 ciclosporin* ***tacrolimus*** *based* ***immunosuppression*** *has* ***resulted*** *in 165 grafts and 192* ***lives*** *being* ***saved*** *during the period 2008 13”*

**Table 5:** Panel B–Physical Sciences

Class and interpretive label	Ten most associated words	Typical sentence
**Class 1** (21.87%): Environmental management & infrastructures	noise, groundwater, hazard, water, local, policy, strategy, arsenic, council, government	*“between 2008 and 2013 ucls ****research ****into ****arsenic ****pollution of ****groundwater ****has had significant impacts on ****policy ****practice and public health ****security ****in ****bangladesh****”*
**Class 2** (19.84%): Advisory & environmental services	TSR, reservoir, storm, course, RS, train, company, service, Ainsa, client	*“the same ****companies ****also use the research to run training ****courses ****for ****employees ****including ****reservoir ****engineers and ****managers ****leading to improved understanding and more informed ****decision ****making about the* ***management ****of hydrocarbon ****reservoirs****”*
**Class 3** (19.78%): Enterprise and innovation	process, design, USD, manufacture, product, company, drug, technology, text, industrial	*“the ****approaches ****also provided evidence that ****platinum ****containing vehicle emission ****catalysts ****are not a source of chloroplatinates in the environment and can therefore continue to be used catalytic ****processes ****underpin* ***production ****in the ****chemicals ****and pharmaceuticals ****industries****“*
**Class 4** (22.8%): Clinical outcomes	patient, clinical, hospital, health, cancer, implant, surgeon, surgery, radon, hip	*“the research has enabled ****neurosurgeons ****to visualise ****white ****matter fibre ****pathways ****which form the communication network of the ****brain ****prior to their ****intervention ****this helps them ****avoid ****cutting these ****fibres ****during the* ***operation ****helping ****patients ****avoid severe cognitive ****deficits ****unrelated to the original ****problem ****that led to the ****surgery****”*
**Class 5** (15.72%): Public engagement and media	talk, public, science, audience, interest, stimulate, view, physic, event, film	*“millions of ****people ****have ****viewed ****television ****contributions ****while tens of thousands have been ****reached ****in theatres and ****science ****fairs with ****positive ****reviews and ****feedback ****confirming a ****stimulation ****of ****public ****interest in and* ***understanding ****of ****chemistry****“*

**Table 6:** Panel C–Social Sciences

Class and interpretive label	Ten most associated words	Typical sentence / other?
**Class 1** (34.14%): Museums and cultural heritage	heritage, museum, London, plan, city, cultural, site, urban, request, conservation	“this has been instrumental in ensuring that **climate** change effects are an intrinsic **part** of management **plans** for historic **sites** commissioned training and research and improved the advice **provided** by commercial **organisations** in the **heritage** sector“
**Class 2** (21.68%): Educational outcomes (children)	child, pupil, study, school, young, girl, age, teacher, family, person	*“and five films were made in* ***pupil*** *referral units for excluded* ***children*** *8 this work which mostly took* ***place*** *in* ***schools*** *and* ***youth*** *centres has led both to increased* ***confidence****”*
**Class 3** (28.01%): Education policy	education, policy, inform, conference, [lall], lambert, mon, guidance, apprenticeship, influence, curriculum	*“beyond its specific* ***influence*** *on examination* ***practice*** *and the* ***publication*** *of* ***examination*** *results the* ***research*** *has achieved significant* ***impacts*** *by stimulating and* ***informing*** *debate among key* ***stakeholders*** *and policymakers in fields* ***related*** *to the provision and regulation of medical* ***education***“
**Class 4** (16.17%): Influencing legal and political processes	committee, wage, common, house, minimum, judicial, reform, tax, constitution, justice	*“****written*** *and* ***oral*** *submissions were also made to the* ***environmental*** *audit select* ***committee*** *eac at the* ***house*** *of* ***commons*** *and the* ***final*** *eac* ***report*** *cited the vibat work substantively and requested that the dft become more progressive on this topic“*

**Table 7:** Panel D–Humanities

Class and interpretive label	Ten most associated words	Typical sentence / other?
**Class 1** (19.96%): European policy	Policy, recommendation, health, EU, budget, innovation, control, Ukraine, support, [edwards], Dutch	*“and one of the major areas of structural* ***funds*** *for new* ***eu*** *member* ***states*** *impact on eu* ***policy*** *on* ***funding*** *research* ***eu*** *support for* ***research*** *and* ***innovation*** *is administered through its* ***framework*** *programmes fps for* ***research*** *and* ***development*** *“*
**Class 2** (17.8%): Media outreach	circulation, radio, coverage, BBC, daily, Guardian, newspaper, readership, press, time	*“mafia* ***brotherhoods*** *was subject to extensive media* ***coverage*** *surrounding its* ***publication*** *in 2011 ensuring the* ***communication*** *of its key insights to a huge audience including* ***interviews*** *on bbc* ***radio*** *5 lives up all* ***night*** *6m* ***listeners*** *per* ***week*** *and newstalk 106 108 fm 12 audience* ***share*** *in* ***dublin****”*
**Class 3** (20.42%): Public engagement, events	public, lecture, event, Jewish, workshop, audience, London, Jews, talk, organise	*“the* ***reach*** *has* ***included*** *diverse* ***audiences*** *in europe the usa australasia and elsewhere it has improved the knowledge and* ***understanding*** *of* ***students*** *and* ***teachers*** *in the uk professionals involved in* ***public*** *history* ***activities*** *in germany and interested* ***members*** *of the* ***public****“*
**Class 4** (11.78%): Culture, history and heritage	archive, heritage, cultural, community, transcribe, researcher, war, Scandinavian, Bentham, travel	*“this has* ***enhanced*** *public* ***awareness*** *of and access to* ***scandinavian*** *literary and cinematic* ***heritage*** *in the uk and* ***internationally*** *produced new* ***cultural*** *resources and transferred* ***skills*** *knowledge and* ***resources*** *between* ***researchers*** *partners in* ***publishing*** *translation”*
**Class 5** (12.3%): Education, language, apps	IPP, app, IGE, site, undergraduate, philosophy, book, Assyrian, religious, student	*“the* ***ige*** *website and ige* ***app*** *remedy this problem by making use of authentic examples* ***sourced*** *from the ice gb* ***corpus*** *to help* ***learners*** *acquire real* ***english*** *the* ***ige*** *website is freely available and has* ***received*** *over 1”*
**Class 5** (17.74%): Art and museums	art, gallery, collection, artist, curator, exhibition, work, display, studiowork, paint	*“the* ***exhibition*** *eva hesse* ***studiowork*** *from 2009 travelled across europe and north* ***america*** *over two years attracting over 200*.*000 visitors it provided* ***cultural*** *enrichment and* ***raised*** *public awareness about how* ***art*** *is made “*

#### Panel A: Life Sciences

The analysis of 122 UCL impact case studies submitted to Panel A produced three distinct classes, or interpretations of type of impact as described in Table 4.

#### Panel B: Physical Sciences

The analysis of 50 UCL impact case studies submitted to Panel B produced five distinct classes, or interpretations of type of impact (Table 5).

#### Panel C: Social Sciences.

he analysis of 75 UCL impact case studies submitted to Panel C produced four distinct classes, or interpretations of type of impact (Table 6).

#### Panel D: Humanities

The analysis of 36 UCL impact case studies submitted to Panel D produced six distinct classes, or interpretations of type of impact (Table 7).

These findings support the conclusion of the Kings study, that different types of impact are common in different disciplines, and that there is a clear relationship between the type of impact and the subject of the UoA. This does not, however appear to preclude the potential for interdisciplinarity as several types of impact are common across UoAs–such as public engagement or impact on policy. Comparing these results to the REF guidance (criteria and working methods) [3] it appears that not all possible areas of impact were explored by all panels. For example, while ‘contributing to the economy’ was included as an example of impact in the guidelines for all of the panels, words associated with such impact (such as ‘company’, ‘Industry’, ‘business’ or ‘pound’) were not significant in any of the classes emerging from the analysis of submissions to Panel A (Life Sciences) nor Panel D (Humanities) and were single words–not the main focus–within Class C1 (Museums and Cultural Heritage) and Class C4 (influencing legal and political processes). On the one hand the absence of economic impact in such key areas is surprising, especially given the significant economic role played by bio-industry and the arts and media industry in the UK. On the other hand, this tendency to focus around topic relevant interpretations of impact is perhaps unsurprising and, as the Kings Study has argued, is possibly a reflection of the research area. We would add that this is also likely to be the result of the process to select case studies, whereby the guidelines set out a need to demonstrate a clear, evidence-based pathway from published research to impact. As such, impact is more likely to be framed and described in terms of the research, rather than from the standpoint of types of impact, reinforcing the connection to the research.

### Impact and research quality

**Fig 2:**
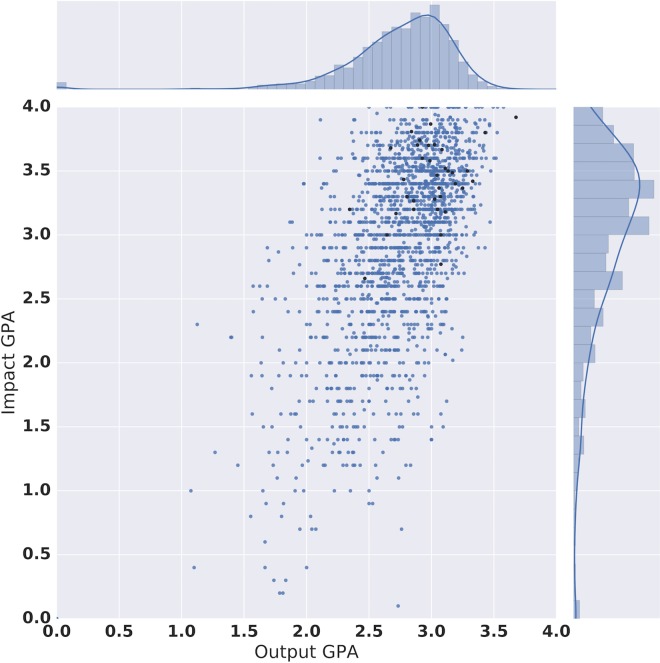
Impact GPA as a function of Output GPA for each Unit of Assessment in REF2014. Sector data is shown in blue, UCL contributions are shown in black. Marginal distributions show the positive skew of the data (Output Median = 2.82, Impact Median = 3.1).

To understand the relationship between impact and research quality, we plotted the impact score (Impact GPA) against the research quality score (Output GPA) for UCL (black) and for the entire database, shown in Fig 2. In terms of the output scores, we compared centres, departments, and research units that submitted to a particular UoA. Plotting the GPA for Impact against the GPA for Output as well as Overall score (not shown) for each UoA in each institution illustrates possible trends and correlations across the sector, with any bias or unusual features in the UCL data (none were found).

From this, it is possible to see that those units of assessment which scored highly in research quality (output GPA) also scored highly in impact. HEFCE guidelines require that research submitted for consideration of Impact be of at least 2* quality; the majority of data is indeed above the 2* level for research outputs (although not all, suggesting that universities are not perfect predictors of their own performance). Indeed Wooding et al (2015) [46] have also found that UK research submitted to the RAE and REF was of better quality than worldwide research on average, arguing that while there is little evidence to support the level of increased quality evident in the REF submissions. This, they argue, suggests that as compared to the previous RAE, the REF results implied a lower citation threshold for declaring a 4*. However, the data show sufficient variation in both Output GPA (1.1–3.68) and Impact GPA (0.1–4.0) for us to argue that there is also evidence of a correlation between output and impact scores (Pearson Product Moment Coefficient = 0.65). A correlation between impact and quality is therefore evident in these results, although we must temper this claim with the previous finding from Derrick and Samuel, which identified a firm belief in an intrinsic link between high ‘societal’ impact and “excellent research” [30] (assumed here to be based on e.g. ranking of journal in which the research evidence is published) in the minds of several REF evaluators prior to the execution of the exercise. The data also exhibits a clustering in the top-right corner of the graph (i.e. an over-representation of higher GPAs). This is likely to be due to the selection process universities employ to present their research in the best possible light. The median and interquartile range (within which 50% of data fall) for Output are 2.8 and 2.6–3.0, and for Impact these figures are 3.1 and 2.6–3.5—demonstrating a high degree of “bunching” around these already high median values.

Given that the data points (Fig 2) are based on Units of Assessment rather than individuals or papers however, it is possible that within an institution there are individuals producing high quality research with limited impact, and others producing research with mediocre academic quality but with high impact. In fact, the REF2014 process did not require the same individuals to be submitted for assessment on quality as those submitted for impact, which has potential consequences for academic career structures and rewards. Nevertheless, by looking at the data in aggregate as Units of Assessment, the overarching picture, we argue that is that there is evidence of a correlation between research quality and impact, which we discuss further below.

## Discussion and Conclusions

This paper set out to understand how researchers and institutions interpreted impact, and how these interpretations affected performance in the REF process. In keeping with previous studies, we have found that impact is interpreted broadly by researchers via impact case studies depicting a range of impact types and beneficiaries (or sectors). By using a methodology that seeks distinctiveness, we have been able to describe six specific categories of impact that researchers have identified in their own work: namely influence on education; public engagement; environmental technologies and solutions; enterprise; policy impact; and clinical applications for the entire REF case study database. While these fit within the examples of impact provided by REF2014, they do not map directly onto them. This, we argue, is both the result of researchers’ and institutions’ interpretations of impact, but also the way that impact was framed, in order to show a direct link to research. Importantly, we found that a number of areas of impact exemplified by the REF guidance, were not evident in our analysis–most notably impact on international development.

The King's Study identified 3,709 unique pathways to impact out of the 6,679 impact cases analysed. In this interpretation fewer than two cases (on average) shared the same pathway to impact. While each impact will be unique, we argue the higher-level categories that we have generated here both encompass the categories described by King’s but also provide a more ‘textured’ picture of what impact means. This more distinct categorization helps clarify the King’s study and, together, the two present a detailed topography of interpretations of impact.

Our findings also provide insight into how research impact was interpreted as a whole and across institutions, and how these interpretations were turned into evidence through the case studies. Examining these case studies in relation to other REF metrics helps address several of the concerns expressed about its addition to the REF exercise [5,6,7,12,13]. It additionally provides methods to compare the categories of impact for an individual institution with the broad categories across the sector, to help scholars and research managers understand how individual institutions are positioning themselves, and capture routes to impact not seen broadly across the sector, but pioneered by specific universities. Similarly, descriptive text analysis on the level of individual institutions could yield a closer reading of language used by different universities; for example, allowing a more nuanced understanding of ‘successful’ submissions, and factors which might favour some universities over others in research assessment processes.

Beyond the researchers’ interpretation of impact our findings indicate that impact within the REF2014 guidelines has been defined, and assessed, broadly [5]. Upon the announcement that impact would form part of the REF2014 exercise, there were concerns about the (lack of) [impact] definitions [29]. Whilst this may have been true, the spread of case studies and the results of their assessment indicate the REF has been able to recognize and reward a diverse range of impact types, with a diverse basis for submission across faculties. Despite the lack of more specific guidance, no single type of impact was more rewarded than others–there is no evidence to suggest that the REF2014 exercise was designed to focus academic research towards commercial or economic goals, for example.

Where there may have been concerns that the political motivations for the REF to measure the value added from research council funding would lead to impact being skewed towards economically-driven outputs, we can see that impact extended well beyond this. A case in point: where it had been found that public engagement “pre-impact” was relegated by some academics as “faddish, superfluous and non-essential” [29] (p. 120, based on 24 interviews with eight vice-chancellors from teaching focused institutions, one principle of performing arts institution, 15 heads of schools at one research intensive institution), we have identified it as forming a central component of impact across the institution within the REF. While the UCL case study indicates that local culture appears to matter (UCL as an institution has heavily invested in diverse manifestations of impact such as public engagement, and supported staff to develop activities–support that appears to have paid off), the importance of public engagement as a form of impact does not appear to be limited to UCL as public engagement was a class found in the analysis of the entire database.

Based on our analysis of impact at the Panel level, we found that certain types of impact are more likely within disciplinary areas, even when the panel guidelines specified wide categories of impact. This in part reflects the specific and local practicalities and social worlds of translational research, as well as the way that the case studies described impact in relation to the research—indeed it makes sense that the impacts in Panel A (life sciences) concentrated on health policy, diagnosis and clinical, it might also suggest that more work is needed to fully legitimise and embed the full range of potential ‘impact work’ available to research staff. Furthermore, it points towards an issue of concern for future research assessment exercises. On one hand, it is clear that a significant part of the UK Government’s motivation for funding research is as a driver for economic growth—the Gov.UK webpage on Science and Innovation (https://www.gov.uk/government/topics/science-and-innovation) states that “The government funds and supports innovation in science, technology and engineering to help the UK’s high-tech industries to thrive” for example). Rewarding all interpretations of impact equally arguably does little to forward this agenda. On the other hand, keeping interpretations of impact open and rewarding all interpretations of impact equally is likely to be important in reassuring researchers that the REF process maintains a sense of academic freedom for researchers. Communicating the evidence that this principle has been enacted in the REF2014 could be a valuable way to build confidence in the process.

Finally, we have identified a positive correlation between research quality and research impact at institutional level, which suggests that there is little evidence that the pursuit of impact detracts from the quality of research. Given the various factors that we have highlighted within the paper, such as selection bias within institutions, which meant that only high quality research was submitted to the REF, we do not want to go too far in interpreting these findings. Future REF exercises will however offer a useful opportunity and data to investigate this relationship further.

Nevertheless, we are aware that our analysis–and the REF process–is unlikely to fully reflect all of the impacts of university research. The REF process made certain requirements that meant that only particular instances of impact–those with a direct line of evidence to ‘two-star’ quality research–were able to be submitted. Further qualitative research with researchers themselves could be valuable in shedding further light on how the REF process itself has shaped how researchers and institutions perceive and operationalise impact.

## Supporting Information

S1 Table REF2014 Units of Assessment. Submissions to the REF were made in 36 units of assessment. An expert sub-panel for each unit of assessment assessed each submission, working under the leadership and guidance of four main panels.
(DOCX)

S2 Table Sector- and institution-specific classes. Comparison table of the impact case study text mining analysis by class for individual institutions and the entire database.
(DOCX)
