# Correction: Beyond Academia – Interrogating Research Impact in the Research Excellence Framework

**DOI:** 10.1371/journal.pone.0172817

**Published:** 2017-02-21

**Authors:** Emma Terama, Melanie Smallman, Simon J. Lock, Charlotte Johnson, Martin Zaltz Austwick

The first author’s name is spelled incorrectly. The correct name is: Emma Terama. The correct citation is: Terama E, Smallman M, Lock SJ, Johnson C, Austwick MZ (2016) Beyond Academia—Interrogating Research Impact in the Research Excellence Framework. PLoS ONE 11(12): e0168533. doi:10.1371/journal.pone.0168533
