# Supplementary material for: Beyond Academia – Interrogating Research Impact in the Research Excellence Framework
Source: PLoS One. 2016 Dec 20;11(12):e0168533. doi: 10.1371/journal.pone.0168533 (PMC5173344; doi:10.1371/journal.pone.0168533)
Supplement: S2 Table — Comparison table of the impact case study text mining analysis by class for individual institutions and the entire database. (DOCX) [file pone.0168533.s002.docx]

**Supporting Information**

**S2 Table. Sector- and institution-specific classes.** Comparison table of the impact case study text mining analysis by class for individual institutions and the entire database.

|  | Entire database | | UCL | Cardiff | ICR | Imperial | King’s | LSHTM | St Georges |  |
| --- | --- | --- | --- | --- | --- | --- | --- | --- | --- | --- |
| Education | Class 1 22.81% | | Class 4 14.43% |  |  |  |  |  |  |  |
|  |  | |  |  |  |  |  |  |  |  |
| Public engage-ment | Class 2 16.96% | | Class 2 16.77% | Class 7 17.17% |  |  | Class 2 29.74% | Class 1 15.45% |  |  |
|  |  | |  |  |  |  |  |  |  |  |
| Enterprise | Class 4 11.8% | | Class 5 21.55% | Class 3 11.02% | Class 1 21.24% | Class 1 37.44% |  |  |  |  |
|  |  | |  |  |  | Class 2 32.79% |  |  |  |  |
|  |  | |  |  |  |  |  |  |  |  |
| Policy | Class 5 17.09% | | Class 1 23.53% | Class 1 17.95% |  |  | Class 1 28.07% | Class 3 26.99% |  |  |
|  |  | |  | Class 5 15.97% |  |  |  |  |  |  |
|  |  | |  |  |  |  |  |  |  |  |
| Environ-mental solutions & energy | Class 3 17.66% | |  | Class 2  11.43% |  |  |  |  |  |  |
|  |  | |  |  |  |  |  |  |  |  |
| Clinical uses | | Class 6  13.68% | Class 3 23.72% | Class 4 10.85% | Class 2 18.58% | Class 3 29.77% | Class 4 13.9% | Class 5 15.77% | Class 1  14.43% | |
|  |  | |  |  | Class 4 18.14% |  |  |  | Class 2 19.59% |  |
|  |  | |  |  | Class 5 23.89% |  |  |  | Class 3 17.53% |  |
|  |  | |  |  |  |  |  |  | Class 4 12.37% |  |
|  |  | |  |  |  |  |  |  |  |  |
| Public health |  | |  | Class 6 15.6% | Class 3 18.14% |  | Class 3 28.29% | Class 2 26.02% | Class 5 18.56% |  |
|  |  | |  |  |  |  |  | Class 4 15.77% | Class 6 17.53% |  |
